# Beta-1 Adrenergic Agonist Treatment Mitigates Negative Changes in Cancellous Bone Microarchitecture and Inhibits Osteocyte Apoptosis during Disuse

**DOI:** 10.1371/journal.pone.0106904

**Published:** 2014-09-11

**Authors:** Joshua M. Swift, Sibyl N. Swift, Matthew R. Allen, Susan A. Bloomfield

**Affiliations:** 1 Departments of Health and Kinesiology, Texas A & M University, College Station, Texas, United States of America; 2 Intercollegiate Faculty of Nutrition, Texas A & M University, College Station, Texas, United States of America; 3 Department of Anatomy and Cell Biology, Indiana University School of Medicine (IUSM), Indianapolis, Indiana, United States of America; Inserm U606 and University Paris Diderot, France

## Abstract

The sympathetic nervous system (SNS) plays an important role in mediating bone remodeling. However, the exact role that beta-1 adrenergic receptors (beta1AR) have in this process has not been elucidated. We have previously demonstrated the ability of dobutamine (DOB), primarily a beta1AR agonist, to inhibit reductions in cancellous bone formation and mitigate disuse-induced loss of bone mass. The purpose of this study was to characterize the independent and combined effects of DOB and hindlimb unloading (HU) on cancellous bone microarchitecture, tissue-level bone cell activity, and osteocyte apoptosis. Male Sprague-Dawley rats, aged 6-mos, were assigned to either normal cage activity (CC) or HU (n = 18/group) for 28 days. Animals were administered either daily DOB (4 mg/kg BW/d) or an equal volume of saline (VEH) (n = 9/gp). Unloading resulted in significantly lower distal femur cancellous BV/TV (−33%), Tb.Th (−11%), and Tb.N (−25%) compared to ambulatory controls (CC-VEH). DOB treatment during HU attenuated these changes in cancellous bone microarchitecture, resulting in greater BV/TV (+29%), Tb.Th (+7%), and Tb.N (+21%) vs. HU-VEH. Distal femur cancellous vBMD (+11%) and total BMC (+8%) were significantly greater in DOB- vs. VEH-treated unloaded rats. Administration of DOB during HU resulted in significantly greater osteoid surface (+158%) and osteoblast surface (+110%) vs. HU-VEH group. Furthermore, Oc.S/BS was significantly greater in HU-DOB (+55%) vs. CC-DOB group. DOB treatment during unloading fully restored bone formation, resulting in significantly greater bone formation rate (+200%) than in HU-VEH rats. HU resulted in an increased percentage of apoptotic cancellous osteocytes (+85%), reduced osteocyte number (−16%), lower percentage of occupied osteocytic lacunae (−30%) as compared to CC-VEH, these parameters were all normalized with DOB treatment. Altogether, these data indicate that beta1AR agonist treatment during disuse mitigates negative changes in cancellous bone microarchitecture and inhibits increases in osteocyte apoptosis.

## Introduction

Osteoporosis is a debilitating skeletal disorder reportedly affecting nearly 44 million in the United States alone [1]. Fragility fractures common in those with advanced osteoporosis can result in a reduced quality of life [2], [3], [4], [5]. Recently, it has been estimated that the cost associated with treating new osteoporotic fractures in the US will total $16.9 billion [6]. Furthermore, a significant number of osteoporotic patients are bedridden, resulting in greater risk for debilitating secondary physiological effects and even death [7].

Similar to the effects of prolonged bed rest in humans, rodent hindlimb unloading (HU) significantly reduces cancellous bone mass and leads to deleterious changes in microarchitecture due to early increases in bone resorption followed by prolonged depressions in bone formation [8], [9], [10], [11]. Unloading-associated reductions in metaphyseal bone mass are associated with increased osteocyte and osteoblast apoptosis. Dramatic increases are observed in the number of apoptotic osteocytes in cancellous bone as early as 3 days after initiation of HU [12], [13]. Isoproterenol, a beta-adrenergic receptor agonist (betaAR) (equally stimulating beta-1 and beta-2 adrenergic receptors), has been found to have anti-apoptotic effects on cultured osteoblasts [14]. Taken together, these data suggest that reducing osteocyte/osteoblast apoptosis during the early stages of unloading may be an effective strategy to preserve cancellous bone mass and maintain osteoblast function.

Although stimulation of the SNS has been documented to increase bone resorption, resulting in reduced cancellous bone mass and microarchitecture, this has been primarily attributed to stimulation of beta-2 adrenergic receptors (beta2AR) [15], [16], [17], [18]. However, the exact role that beta-1 adrenergic receptors (beta1AR) have in this process has not been elucidated. Dobutamine (DOB), primarily an beta1AR agonist, significantly blunts HU-induced reductions in cortical bone area and cross-sectional moment of inertia (CSMI), as well as mitigating the decreases in femoral mid-diaphyseal cortical bone formation [19]. Furthermore, we have previously demonstrated the ability of DOB to inhibit reductions in cancellous bone formation and mitigate losses in bone mass at the proximal tibia and femoral neck [20]. The data presented in the current paper derive from a similar but separate protocol and demonstrate a direct relationship between alterations in osteoblast activity, osteocyte apoptosis, and cancellous microarchitecture resulting from beta1AR agonist treatment during hindlimb unloading. These results provide useful insight into underlying mechanisms involved in disuse-induced bone loss.

Hence, the purpose of the current project was to extend our previous findings and characterize the independent and combined effects of DOB and hindlimb unloading on cancellous bone microarchitecture, osteoblast activity, and osteocyte apoptosis. We hypothesized that unloading would increase osteocyte apoptosis in animals experiencing metaphyseal bone loss and that DOB administered during 28-day HU would mitigate deleterious changes in cancellous bone microarchitecture and increase osteocyte cell survival.

## Methods

### Ethics Statement

All research was conducted in a facility accredited by the Association for the Assessment and Accreditation of Laboratory Animal Care (AAALAC). All procedures involving animals were reviewed and approved by the Texas A&M University Institutional Animal Care and Use Committee. At the end of the study, animals were anesthetized with a ketamine-xylazine cocktail (ketamine 50 mg/kg, medetomidine 0.5 mg/kg) and subsequently euthanized by decapitation, in accordance with the recommendations and guidelines of the American Veterinary Medical Association.

### Animals and Experimental Design

Thirty-six male Sprague-Dawley rats were obtained from Harlan (Houston, TX) at 6 months of age and allowed to acclimate to their surroundings for 14 days prior to initiation of the study. All animals were housed in a temperature-controlled (23±2°C) room with a 12-hour light-dark cycle in an AAALAC-accredited animal care facility and were provided standard rodent chow (Harlan Teklad 8604) and water ad-libitum.

All animals were randomly assigned to one of two activity groups according to body mass on day -1: normal ambulatory cage activity (CC; n = 18) or hindlimb unloading (HU; n = 18) for 28 days. Each activity group was further divided by random assignment to receive daily intraperitoneal (IP) injections of dobutamine (DOB; 4 mg/kg body mass n = 9) or an equal volume of saline solution (VEH; n = 9). We previously demonstrated that this dose of DOB effectively mitigated disuse-induced reductions in proximal tibia total vBMD during 28-day HU [20]. Dobutamine hydrochloride solution (Sigma-Aldrich Corp.) was made fresh daily and stored, along with saline solution, at 4°C until usage.

### Hindlimb Unloading

Hindlimb unloading was achieved by tail suspension as previously described [10], [21]. Briefly, while the rat was under anesthesia, the tail was cleaned and dried thoroughly. A thin layer of adhesive (Amazing Goop, Eclectic Products, LA) was applied to the proximal half of the tail along the medial and lateral sides. A standard porous tape (Kendall, Mansfield, MA) harness was pressed firmly to the glue and allowed to dry (∼30 min). A paper clip was used to attach the animal’s tail harness to a swivel apparatus on the wire spanning the top of an 18”×18”×18” cage.

Calcein injections (25 mg/kg body mass) were given subcutaneously 9 and 2 days prior to euthanasia to label mineralizing bone for histomorphometric analyses. HU animals were anesthetized before removal from tail suspension at the end of the study to prevent any weight bearing by the hindlimbs. At necropsy, left femora were removed, cleaned of soft tissue, and stored at 4°C in 70% ethanol for *ex vivo* μCT and pQCT scans and subsequent histomorphometry. Distal right femora were stored in paraformaldehyde for paraffin embedding.

### Ex Vivo Micro Computed Tomography (μCT)

Microarchitecture of cancellous bone located in the distal left femur was determined using a Skyscan 1172 high-resolution desk-top micro-computed tomography system. Bones were wrapped in parafilm to prevent drying during the scanning. Scans were obtained using an x-ray source set at 60 kV and 167 µA over an angular range of 180 degrees (rotational steps of 0.70 degrees) with a 12 µm pixel size. Projection images were reconstructed using standard Skyscan software. The trabecular bone compartment was segmented from the cortical shell for 50 slices in a region ∼0.5 mm above the most proximal portion of the growth plate for each animal. Outcomes variables include cancellous bone volume/tissue volume (BV/TV, %), trabecular number (Tb.N, µm^−1^), and trabecular thickness (Tb.Th, µm).

### Ex Vivo Peripheral Quantitative Computed Tomography (pQCT)

Scans were performed *ex vivo* at the distal metaphysis of the left femur with a Stratec XCT Research-M device (Norland Corp., Fort Atkinson, WI), using a voxel size of 70 µm and a scanning beam thickness of 500 µm. Daily calibration of this machine was performed with a hydroxyapatitie standard cone phantom. Femora were placed in a 70% ethanol filled vial during the course of the scan. Transverse images of the right femur were taken at 4.5, 5.0, 5.5, and 6.0 mm from the distal femur plateau. A standardized analysis for metaphyseal bone (contour mode 3, peel mode 2, outer threshold of 0.214 g/cm^3^, inner threshold of 0.605 g/cm^3^) was applied to each section. Values of total and cancellous volumetric bone mineral density (vBMD), total bone mineral content (BMC) and total bone area were averaged across the three slices to yield a mean value for each animal.

### Histomorphometry Analysis

Undemineralized distal left femur were subjected to serial dehydration and embedded in methylmethacrylate (Aldrich M5, 590-9). Serial frontal sections were cut 8 µm thick and left unstained for fluorochrome label measurements. Additionally, 4 µm thick sections treated with von Kossa staining were used for measurement of cancellous bone volume normalized to tissue volume (BV/TV), and osteoid (Os/BS), osteoblast (ObS/BS), and osteoclast (OcS/BS) surfaces as a percent of total cancellous surface. Osteoclasts were conservatively defined as cells adhering to the bone surface with at least 2 nuclei and a foamy cytoplasm (as determined from tetrachrome counterstain). Adipocyte density was calculated as number of adipocytes (Ad.N) divided by the marrow area (Ma.Ar) of the region of measurement. The histomorphometric analyses were performed by using the OsteoMeasure Analysis System, Version 1.3 (OsteoMetrics, Atlanta, GA). A defined region of interest was established ∼1 mm from the growth plate and within the endocortical edges encompassing 8−9 mm^2^ at ×40 magnification. Total bone surface (BS), single labeled surface (sLS), double-labeled surface (dLS), interlabel distances, bone volume, and osteoid/osteoclast/osteoblast surfaces were measured at ×200 magnification. Mineral apposition rate (MAR, μm/day) was calculated by dividing the average interlabel width by the time between labels (7 days), and mineralizing surface (MS) for cancellous bone surfaces (BS) was calculated by using the formula %MS/BS  =  {[(sLS/2) + dLS]/BS} X 100. Bone formation rate (BFR) was calculated as (MAR x MS/BS). All nomenclature for cancellous histomorphometry follows standard usage [22].

### Osteocyte Apoptosis and Occupied Lacunae

In situ osteocyte apoptosis was assessed from distal femur sections as previously described [23]. In brief, distal left femurs were decalcified and subsequently embedded in paraffin, and serial frontal sections were cut 8 µm thick and mounted on slides. Apoptosis of osteocytes was detected by in situ terminal deoxynucleotidyl transferase dUTP nick end labeling (TUNEL) using the DNA fragmentation TdT enzyme and fluorescein-dUTP label (Roche Diagnostics Corp., Indianapolis, IN, USA) in distal femoral sections counterstained with hematoxylin QS (Vector Laboratories). Osteocytes residing in trabeculae within an 8−9 mm^2^ region of interest 1 mm distal to the growth plate were quantified using the OsteoMeasure Analysis System, Version 1.3 (OsteoMetrics). The total number of osteocytes (N.Ot) within the region was first counted (under normal light), followed by identification of TUNEL^+^ osteocytes using ultraviolet light at ×200 magnification. The percentage of apoptotic osteocytes was calculated as (TUNEL^+^ Ot/total N.Ot) × 100. The total number of lacunae within this same region of interest was also quantified (under normal light) to determine the total number of osteocytic lacunae (N.Ot/B.Ar) and osteocyte occupancy frequency (%; total N.Ot/total # lacunae).

### Statistical Analyses

All data were expressed as means ± SEM, and their statistical relationships were evaluated using the statistical package SPSS (v.15) and were analyzed using a two-factor ANOVA (drug and gravity). When a significant main effect was found, Tukey’s post-hoc analyses were performed for pairwise comparisons. For all data, statistical significance was accepted at p≤0.05.

## Results

### Dobutamine administration attenuates early reductions in body mass associated with disuse

**Figure 1:**
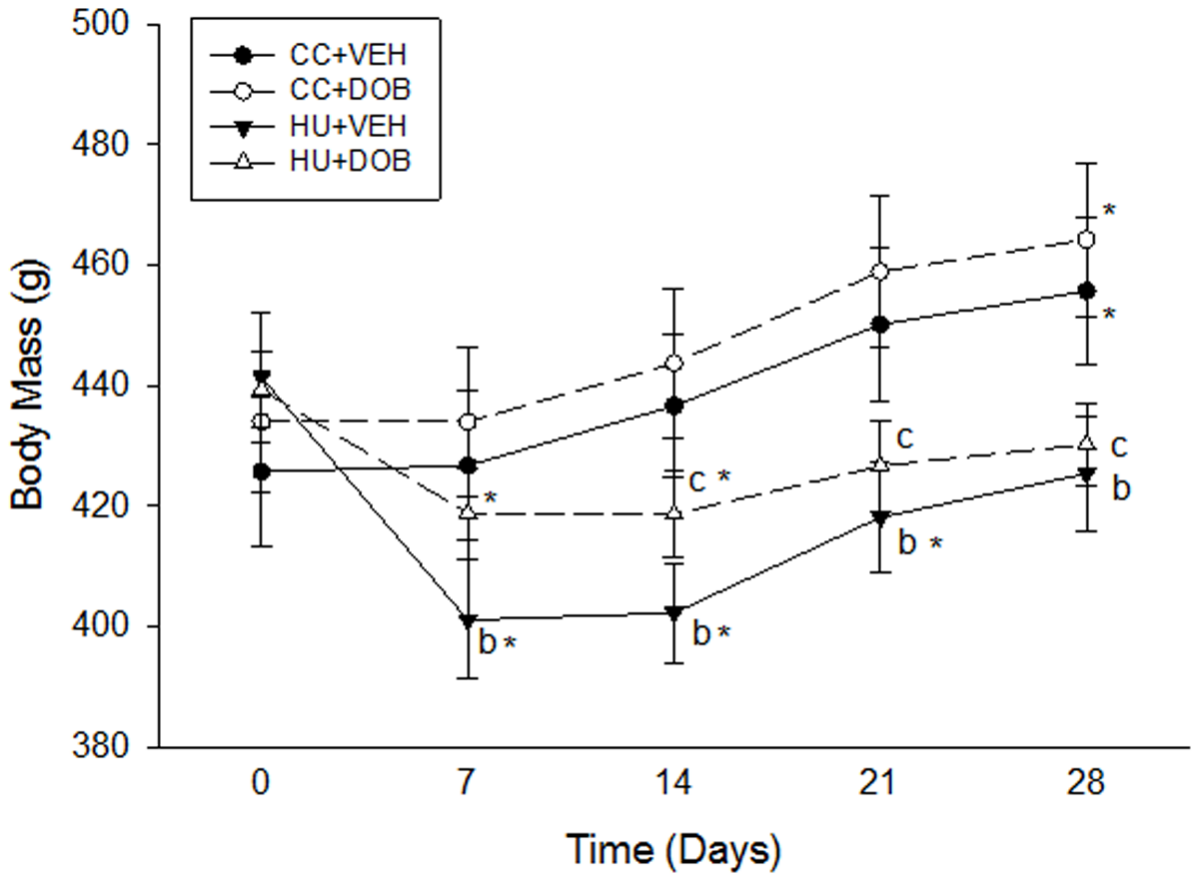
Effects of dobutamine (DOB) or vehicle (VEH) administration during hindlimb unloading (HU) or ambulatory cage activity (CC) on body mass. * vs. Day 0 (p≤0.05); ^a^HU+DOB vs. HU+VEH (p≤0.05); ^b^HU+VEH vs. CC+VEH (p≤0.05); ^c^HU+DOB vs. CC+DOB (p≤0.05).

Hindlimb unloading resulted in early reductions in body mass by Day 7 (−9% vs. Day 0), which continued through Day 21 (−5% vs. Day 0; **Fig. 1**) and recovered to baseline levels by Day 28. Although DOB treatment during unloading did not inhibit early losses in body mass (Day 7/14: −7% vs. Day 0), it did attenuate this reduction (+4% vs. HU-VEH). Furthermore, recovery of total body mass in HU-DOB animals occurred by Day 21 and continued through Day 28. Both groups of cage controls similarly gained body mass over the experimental period with no difference between treatments.

### Reductions in metaphyseal bone microarchitecture and mass during unloading are mitigated with DOB treatment

**Figure 2:**
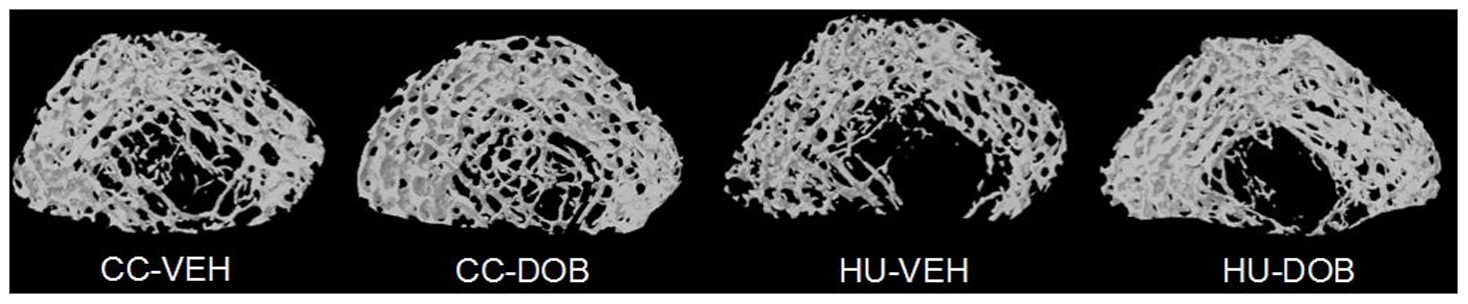
Representative three-dimensional mCT images of the distal femur metaphysis cancellous bone in dobutamine- (DOB) or vehicle- (VEH) treated rodents during hindlimb unloading (HU) or ambulatory cage activity (CC).

To assess changes in cancellous bone microarchitecture, *ex vivo* µCT scans were performed on the distal femur. Unloading resulted in significantly lower BV/TV (−33%), Tb.Th (−11%), and Tb.N (−25%) compared to ambulatory controls (CC-VEH) (**Table 1** **,** **Fig. 2**). DOB treatment during disuse attenuated these differences in cancellous bone microarchitecture. BV/TV (+29%), Tb.Th (+7%), and Tb.N (+21%) were significantly greater in DOB-treated vs. VEH-treated animals subjected to disuse. There was no effect of DOB treatment on trabecular bone microarchitecture in weightbearing control rats.

**Table 1:** Effects of dobutamine (DOB) or vehicle (VEH) administration during hindlimb unloading (HU) or ambulatory cage activity (CC) on distal femur cancellous bone microarchitecture and structure as measured by *ex vivo* microCT scans.

	CC		HU	
	VEH	DOB	VEH	DOB
BV/TV (%)	20.23±1.36	21.05±1.24	13.46±0.74^b^	17.42±0.96^a^ ^,c^
Tb.Th (µm)	93.10±2.09	96.04±1.39	83.36±1.95^b^	89.08±1.91^a^ ^,c^
Tb.N (mm^−1^)	2.16±0.12	2.19±0.11	1.61±0.08^b^	1.95±0.09^a^

a HU+DOB vs. HU+VEH (p≤0.05); ^b^HU+VEH vs. CC+VEH (p≤0.05); ^c^HU+DOB vs. CC+DOB (p≤0.05).

Hindlimb unloading resulted in significantly lower total BMC (−17%) and vBMD (−11%) and cancellous vBMD (−25%) as compared to ambulatory controls (CC-VEH; **Table 2**). DOB administration during disuse mitigated reductions in two of these parameters, resulting in greater total BMC (+8%) cancellous vBMD (+11%) compared to VEH-treated animals subjected to disuse (HU-VEH). There was no effect of either unloading or DOB on proximal tibia bone area. DOB administration did not significantly alter any parameters in weightbearing cage control animals.

**Table 2:** Effects of dobutamine (DOB) or vehicle (VEH) administration during hindlimb unloading (HU) or ambulatory cage activity (CC) on metaphyseal bone mass and geometry at the distal femur as measured by *ex vivo* pQCT scans.

	CC		HU	
	VEH	DOB	VEH	DOB
Total BMC (mg)	12.19±0.42	12.03±0.29	10.10±0.25^b^	10.87±0.27^a^ ^,c^
Total Bone Area (mm^2^)	20.61±0.76	20.25±0.40	19.18±0.61	20.49±0.54
Total vBMD (mg/cm^3^)	593.33±11.33	595.20±11.58	531.02±16.39^b^	533.32±11.26^c^
Cancellous vBMD (mg/cm^3^)	329.29±15.27	320.25±12.12	245.96±7.77^b^	271.85±6.50^a^ ^,c^

a HU+DOB vs. HU+VEH (p≤0.05); ^b^HU+VEH vs. CC+VEH (p≤0.05); ^c^HU+DOB vs. CC+DOB (p≤0.05).

### Dobutamine administration during disuse inhibits reductions in bone formation by maintaining osteoblast surface

**Figure 3:**
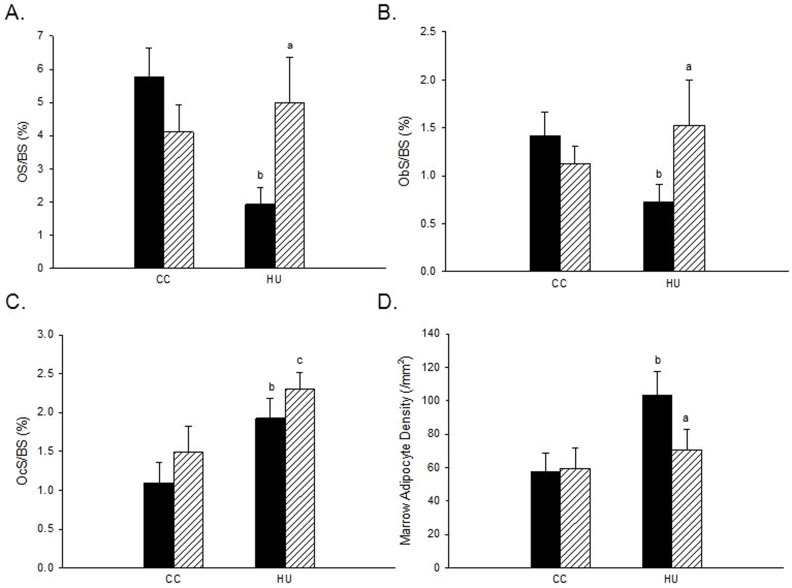
Effects of dobutamine (DOB) or vehicle (VEH) administration during hindlimb unloading (HU) or ambulatory cage activity (CC) on cancellous bone measures of histomorphometry. *A*: Osteoid Surface (OS/BS). *B*: Osteoblast Surface (ObS/BS). *C*: Osteoclast Surface (OcS/BS). *D*: Adipocyte Density (N.Ad/Ma.Ar). VEH groups are represented by black bars; DOB groups are represented by striped bars. ^a^HU+DOB vs. HU+VEH (p≤0.05); ^b^HU+VEH vs. CC+VEH (p≤0.05); ^c^HU+DOB vs. CC+DOB (p≤0.05).

Hindlimb unloading induced significantly lower OS/BS (−66%) and Ob.S/BS (−49%), and greater Oc.S/BS (+75%) and adipocyte density (+81%) within bone marrow as compared to ambulatory controls (**Fig. 3** **,** **4**). DOB treatment during HU resulted in significantly greater OS/BS (+158%) and Ob.S/BS (+110%), and reduced adipocyte density (−32%) vs. VEH-treated animals subjected to HU. Furthermore, Oc.S/BS was significantly greater in HU-DOB (+55%) vs. CC-DOB group (**Fig. 3C**). DOB administration did not affect any of these parameters in weightbearing cage controls.

**Figure 4:**
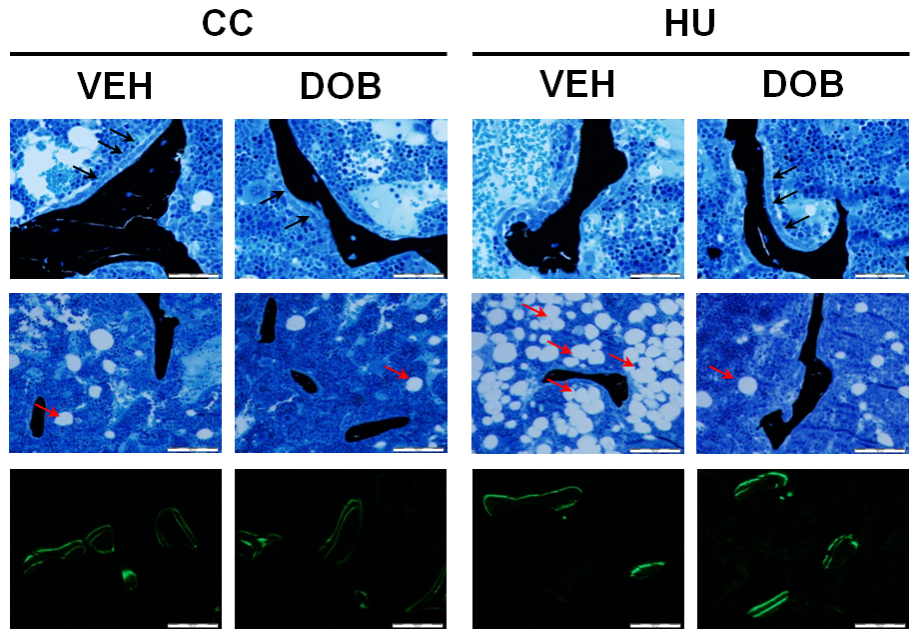
Effects of dobutamine (DOB) or vehicle (VEH) administration during hindlimb unloading (HU) or ambulatory cage activity (CC) on cancellous bone bone cell activity and marrow adipocyte proliferation. (*Top Row*) Representative micrographs of osteoblasts (von Kossa and tetrachrome stain; ×400 magnification), (*Middle Row*) marrow adipocytes (x200 magnification), and (*Bottom Row*) fluorochrome labeling (calcein, 200×) on the surface of cancellous bone at the distal femur. Note the increased number of osteoblasts (*black arrows, top row*) and greater osteoid surface (*below osteoblasts*) in the HU+DOB group vs. HU+VEH. Also, note the increased number of marrow adipocytes (*red arrows, middle row*) in the HU+VEH group. The increased number of osteoblasts noted in the von Kossa and tetrachrome stained slides from HU+DOB samples correlated with extensive flurochrome labeling and large interlabel width (*bottom row*).

Hindlimb unloading resulted in lower MS/BS (−52%), MAR (−30%), and cancellous BFR (−67%) as compared to ambulatory controls (**Fig. 4** **,** **5**). DOB treatment during unloading resulted in significantly greater MS/BS (+140%), MAR (+55%), and BFR (+200%) than in vehicle-treated unloaded rats (HU-VEH). DOB administration did not affect dynamic histomorphometry measures of bone formation activity in weightbearing cage controls.

**Figure 5:**
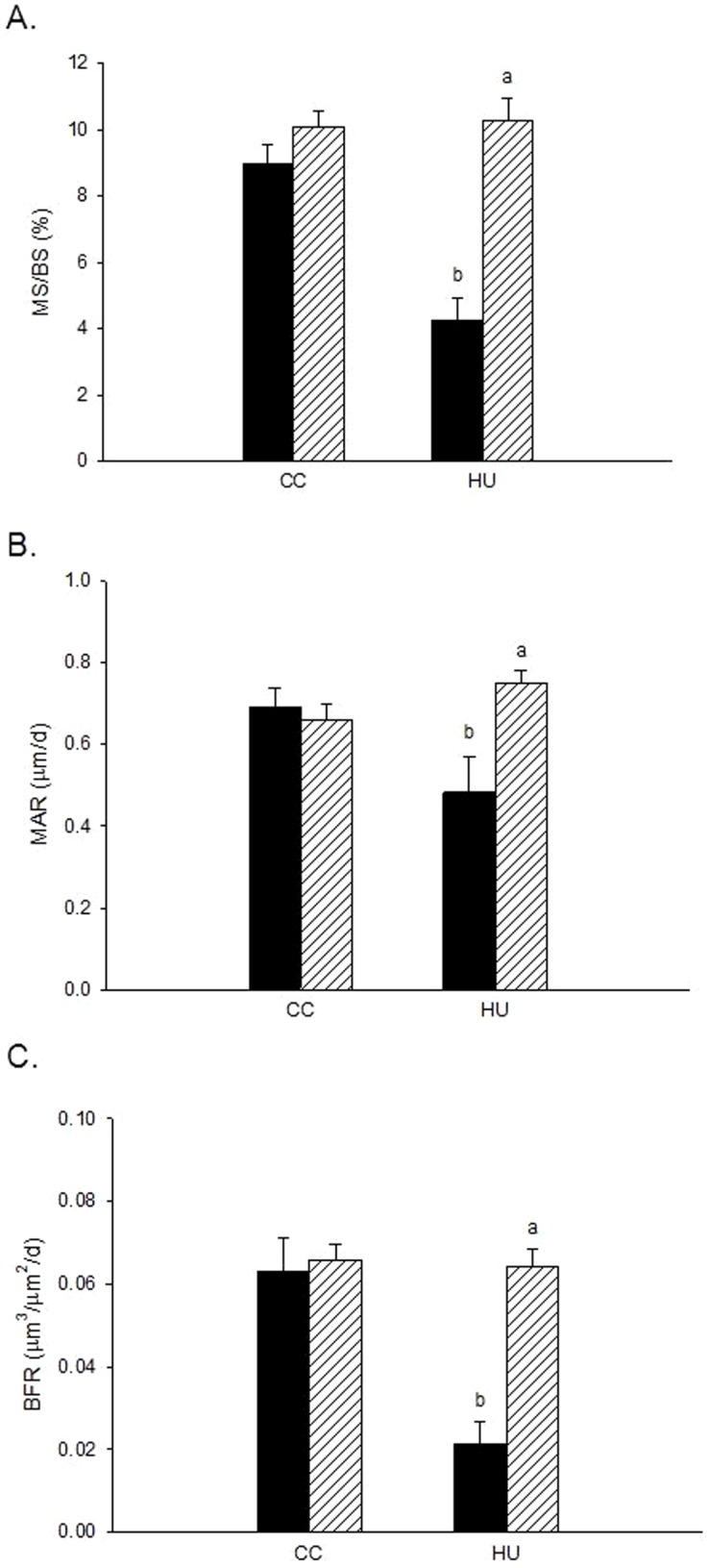
Effects of dobutamine (DOB) or vehicle (VEH) administration during hindlimb unloading (HU) or ambulatory cage activity (CC) cancellous bone dynamic histomorphometry analyses measured at the distal femur. *A*: Mineralizing Surface (%MS/BS). *B*: Mineral Apposition Rate (MAR). *C*: Bone Formation Rate (BFR). VEH groups are represented by black bars; DOB groups are represented by striped bars. ^a^HU+DOB vs. HU+VEH (p≤0.05); ^b^HU+VEH vs. CC+VEH (p≤0.05); ^c^HU+DOB vs. CC+DOB (p≤0.05).

### Increased osteocyte apoptosis and reduced osteocytic lacunae evidenced during rodent hindlimb unloading is abolished with dobutamine treatment

**Figure 6:**
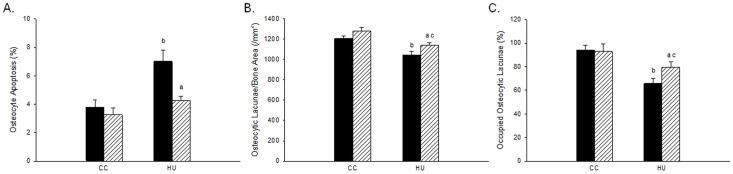
Effects of dobutamine (DOB) or vehicle (VEH) administration during hindlimb unloading (HU) or ambulatory cage activity (CC) on cancellous bone osteocytes measured at the distal femur. *A*: TUNEL+ osteocytes (%). *B*: Total number of osteocytic lacunae (N.Ot/B.Ar). *C*: Osteocyte occupancy frequency (%). VEH groups are represented by black bars; DOB groups are represented by striped bars. ^a^HU+DOB vs. HU+VEH (p≤0.05); ^b^HU+VEH vs. CC+VEH (p≤0.05); ^c^HU+DOB vs. CC+DOB (p≤0.05).

*In situ* nick-end labeling was used to determine the prevalence of osteocyte apoptosis within distal femur cancellous bone in rodents subjected to HU and/or beta1AR agonist treatment. Unloading resulted in a significantly greater percentage of apoptotic cancellous osteocytes (+85%) as compared to ambulatory controls (**Fig. 6A**). DOB-treatment during disuse prevented this increase in osteocyte apoptosis, which was 36% lower than in vehicle-treated rats. There was no difference in osteocyte apoptosis between the ambulatory control groups.

Hindlimb unloading resulted in reduced osteocyte number (−16%) and frequency (−30%) compared to CC-VEH animals (**Fig. 6** **B–C**). Animals treated with DOB during disuse mitigated these reductions, resulting in an increased number of cancellous bone osteocytes per bone area (+9%) and percent of occupied osteocytic lacunae (+21%) compared to VEH-treated HU rats.

## Discussion

Our data confirm that dobutamine treatment during unloading inhibited reductions in cancellous bone formation, resulting in protection of cancellous bone mineral density (vBMD) and bone volume (BV/TV). Furthermore, dobutamine treatment prevented increased cancellous osteocyte apoptosis during disuse. We found no effect of DOB treatment on any of these outcomes when it was administered to rats experiencing normal ambulatory cage activity. Taken together, these data demonstrate the dynamic role that beta-1 adrenergic agonist signaling has on maintaining cancellous bone during periods of reduced weightbearing or unloading.

Data from this experiment demonstrate higher forming and similar resorbing surfaces with dobutamine treatment during unloading relative to unloading controls. DOB administration resulted in significantly greater osteoblast (+110%) and osteoid (2.6-fold) surface within cancellous bone of the distal femur as compared to vehicle-treated hindlimb unloaded rodents (**Fig. 3** **,** **4**). Most strikingly, these increases in osteoblast surface were accompanied by 140% to 200% greater mineralizing surface and bone formation rate (**Fig. 4** **,** **5**) and 55% greater mineral apposition rate vs. HU-VEH group. Additionally, dobutamine treatment during unloading resulted in significantly greater osteoclast surface (+110%) compared to vehicle treated ambulatory controls (**Fig. 3**). These data indicate that specific stimulation of β-1 adrenoreceptors in the setting of disuse is altering remodeling through a mechanism of stimulating both osteoclasts and osteoblast activity, but affecting osteoblast activity (MAR) enough to maintain bone balance and therefore mitigate reductions in bone mass.

Previous work has demonstrated that salbutamol, a β-2 adrenoreceptor agonist, administered to OVX rodents did not significantly alter osteoblast or osteoclast activity as compared to untreated estrogen deficient rats [18]. However, salbutamol-treated OVX animals significantly increased both active bone resorbing and forming surfaces as compared to sham-controls. Mice administered isoproterenol (equally stimulating β-1 and β-2 adrenergic receptors) during unloading exhibit significant reductions in cancellous BV/TV, attributable to significantly lowered bone formation [24]. Numerous investigations have demonstrated that beta2AR agonist administration in weightbearing rats reduces net bone formation by inhibiting osteoblast and increasing osteoclast differentiation. Takeda et al. [25] showed that isoproterenol, a non-specific β-adrenergic agonist, decreased cancellous BV/TV by significantly reducing osteoblast number and bone formation rate (−24–42% vs. controls). Furthermore, salbutamol treatment induced significant reductions in both tibia and femur cancellous bone volume as a consequence of significantly increased osteoclast surface [15]. Deletion of all adrenergic receptors in skeletally mature mice results in even greater reductions in distal femur bone formation (−63% vs wild-type littermates) [26]. Taken together, these data suggest opposing roles of beta1AR and beta2AR on osteoblasts, and that, when activated, beta2AR inhibit osteoblast activity, whereas signaling via beta1AR promotes bone formation.

Administration of β-2 adrenergic agonists results in significant and deleterious modifications to cancellous bone microarchitecture. Both salbutamol and clenbuterol treatment significantly reduces metaphyseal bone volume by reducing Tb.Th and increasing Tb.Sp, resulting in increased rodlike trabecular structures [15], [16], [17]. Trabecular microarchitecture was similarly affected by concurrent beta2AR administration in OVX rats [18]. Data from our current investigation provide evidence for an opposing effect of dobutamine treatment in the context of disuse. Dobutamine administration significantly mitigated unloading associated reductions in BV/TV by attenuating reduced Tb.Th and Tb.N (**Table 1**). In contrast to the aforementioned deleterious effects of β-2 adrenergic agonists, dobutamine administration to rodents engaged in normal cage activity did not produce any significant alterations in cancellous bone microarchitecture.

Reductions in metaphyseal bone mass and structure during hindlimb unloading have been characterized by immediate increases in osteocyte and osteoblast apoptosis. Recent data have demonstrated a significant increase in cancellous osteocyte apoptosis in unweighted proximal tibiae by day 3 of HU, persisting for up to 18 days after disuse [12], [13], [27]. An in vitro study using isoproterenol, an Adrb agonist (equally stimulating beta1AR and beta2AR), demonstrated beta-adrenergic stimulation to have anti-apoptotic effects on cultured osteoblasts [14]. Data from our investigation provides further evidence that beta1AR-signaling protects osteocytes from apoptosis, as dobutamine treatment during unloading inhibited disuse-induced increases in osteocyte apoptosis and reductions in osteocyte number and frequency in cancellous bone as compared to HU-VEH rodents (**Fig. 6**). These data provide preliminary evidence for the role of beta-1 adrenergic signaling to inhibit apoptosis of mechano-sensing cancellous osteocytes during disuse.

Genetic mouse models have demonstrated the potentially unique effects of β-1 and β-2 adrenergic receptors on osteoclasts and osteoblasts, ultimately affecting cancellous bone mass and microarchitecture. Beta2AR and beta1AR knock-out (KO) mice have demonstrated a high and low bone mass phenotype, respectively, whereas beta1/2AR receptor double-KO mice exhibit a marked reduction in cancellous BFR vs. wild types [28], [29]. Furthermore, complete deletion of all 3 Adrb receptors results in a significant reduction in bone formation, although trabecular microarchitecture and bone volume remain unchanged [26]. Pierroz and colleagues [29] demonstrated that beta1AR receptor-deficient mice do not respond to mechanical loading, whereas beta2AR KO mice and wild-type littermates were found to respond normally. Taken together, these data suggest that the higher bone mass phenotype in beta2AR KO mice may be due to enhanced β-1 adrenergic receptor activity, stimulating bone formation in the absence of the inhibitory effects of β-2 adrenergic receptors on osteoblasts. Our results are in agreement with these genetic mouse studies, demonstrating distinct, opposing effects of beta1AR and beta2AR on bone remodeling, especially during a period of disuse. Delineating the effects of beta1AR stimulation on disuse-sensitive cancellous bone during reduced mechanical loading is crucial to defining the underlying mechanisms responsible for bone loss.

One limitation to the current study was the lack of available serum to determine whether beta1AR treatment mitigated the usual decline in circulating insulin-like growth factor 1 (IGF-1) during disuse. Complicating this picture is the fact that hindlimb unloading induces resistance to IGF-1 by inhibiting activation of the IGF-1 signaling pathways [30], [31]. Furthermore, serum IGF-1 is lower in beta1AR and beta2AR knock-out mice vs. wild-type, and femur BMD and cancellous BV/TV in beta1/2AR KO mice has been associated with reduced systemic IGF-1 [29]. We speculate that IGF-1 signaling likely played a critical role in bone’s response to beta1AR agonist treatment during disuse. A second limitation is the lack of immunohistochemistry analyses of beta1AR and beta2AR expression in osteoblasts and osteoclasts within weightbearing bone in this study. Previous investigations have determined that human periosteal and rodent osteoblasts express both beta1AR and beta1AR, although the majority of research demonstrates beta2AR to be the primary receptor expressed in osteoblasts [25], [32], [33], [34], [35]. However, to our knowledge it has yet to be determined whether beta1AR are expressed in osteocytes. Understanding how disuse may alter expression of these receptors in bone cells, as well as the mechanisms responsible for the skeletal effects of beta1AR agonist stimulation, is a critical next step in this line of research.

In conclusion, these data contribute significant evidence to the importance of β-1 adrenergic receptor agonist signaling in bone to mitigate disuse-induced increases in bone fragility and reductions in bone mass. Specifically, treatment with beta1AR agonist during unloading significantly impacted metaphyseal bone and inhibited associated increases in osteocyte apoptosis. These data further define the role that β-adrenergic receptors have in mediating bone’s response to periods of disuse or unloading.

## References

[1] (2002) America's bone health: the state of osteoporosis and low bone mass in our nation. Washington, DC: National Osteoporosis Foundation.

[2] Adachi JD, Loannidis G, Berger C, Joseph L, Papaioannou A, et al (2001) The influence of osteoporotic fractures on health-related quality of life in community-dwelling men and women across Canada. Osteoporos Int 12: 903–908. 11804016 10.1007/s001980170017

[3] Cooper C (1997) The crippling consequences of fractures and their impact on quality of life. Am J Med 103: 12S–17S discussion 17S–19S. 10.1016/s0002-9343(97)90022-x 9302893

[4] Ensrud KE, Thompson DE, Cauley JA, Nevitt MC, Kado DM, et al (2000) Prevalent vertebral deformities predict mortality and hospitalization in older women with low bone mass. Fracture Intervention Trial Research Group. J Am Geriatr Soc 48: 241–249. 10733048 10.1111/j.1532-5415.2000.tb02641.x

[5] Oleksik A, Lips P, Dawson A, Minshall ME, Shen W, et al (2000) Health-related quality of life in postmenopausal women with low BMD with or without prevalent vertebral fractures. J Bone Miner Res 15: 1384–1392. 10893688 10.1359/jbmr.2000.15.7.1384

[6] Burge R, Dawson-Hughes B, Solomon DH, Wong JB, King A, et al (2007) Incidence and economic burden of osteoporosis-related fractures in the United States, 2005–2025. J Bone Miner Res 22: 465–475. 17144789 10.1359/jbmr.061113

[7] Ehrlich PJ, Lanyon LE (2002) Mechanical strain and bone cell function: a review. Osteoporos Int 13: 688–700. 12195532 10.1007/s001980200095

[8] Allen MR, Bloomfield SA (2003) Hindlimb unloading has a greater effect on cortical compared with cancellous bone in mature female rats. J Appl Physiol 94: 642–650. 12391029 10.1152/japplphysiol.00656.2002

[9] Baek K, Barlow AA, Allen MR, Bloomfield SA (2008) Food restriction and simulated microgravity: effects on bone and serum leptin. J Appl Physiol 104: 1086–1093. 18276897 10.1152/japplphysiol.01209.2007

[10] Bloomfield SA, Allen MR, Hogan HA, Delp MD (2002) Site- and compartment-specific changes in bone with hindlimb unloading in mature adult rats. Bone 31: 149–157. 12110428 10.1016/s8756-3282(02)00785-8

[11] Turner RT, Lotinun S, Hefferan TE, Morey-Holton E (2006) Disuse in adult male rats attenuates the bone anabolic response to a therapeutic dose of parathyroid hormone. J Appl Physiol 101: 881–886. 16675609 10.1152/japplphysiol.01622.2005

[12] Aguirre JI, Plotkin LI, Stewart SA, Weinstein RS, Parfitt AM, et al (2006) Osteocyte apoptosis is induced by weightlessness in mice and precedes osteoclast recruitment and bone loss. J Bone Miner Res 21: 605–615. 16598381 10.1359/jbmr.060107

[13] Basso N, Heersche JN (2006) Effects of hind limb unloading and reloading on nitric oxide synthase expression and apoptosis of osteocytes and chondrocytes. Bone 39: 807–814. 16765658 10.1016/j.bone.2006.04.014

[14] Chen X, Song IH, Dennis JE, Greenfield EM (2007) Endogenous PKI gamma limits the duration of the anti-apoptotic effects of PTH and beta-adrenergic agonists in osteoblasts. J Bone Miner Res 22: 656–664. 17266398 10.1359/jbmr.070122

[15] Bonnet N, Benhamou CL, Beaupied H, Laroche N, Vico L, et al (2007) Doping dose of salbutamol and exercise: deleterious effect on cancellous and cortical bones in adult rats. J Appl Physiol 102: 1502–1509. 17185495 10.1152/japplphysiol.00815.2006

[16] Bonnet N, Benhamou CL, Brunet-Imbault B, Arlettaz A, Horcajada MN, et al (2005) Severe bone alterations under beta2 agonist treatments: bone mass, microarchitecture and strength analyses in female rats. Bone 37: 622–633. 16157516 10.1016/j.bone.2005.07.012

[17] Bonnet N, Brunet-Imbault B, Arlettaz A, Horcajada MN, Collomp K, et al (2005) Alteration of trabecular bone under chronic beta2 agonists treatment. Med Sci Sports Exerc 37: 1493–1501. 16177600 10.1249/01.mss.0000177592.82507.95

[18] Bonnet N, Laroche N, Beaupied H, Vico L, Dolleans E, et al (2007) Doping dose of salbutamol and exercise training: impact on the skeleton of ovariectomized rats. J Appl Physiol 103: 524–533. 17478603 10.1152/japplphysiol.01319.2006

[19] Bloomfield SA, Girten BE, Weisbrode SE (1997) Effects of vigorous exercise training and beta-agonist administration on bone response to hindlimb suspension. J Appl Physiol 83: 172–178. 9216961 10.1152/jappl.1997.83.1.172

[20] Swift JM, Hogan HA, Bloomfield SA (2013) Beta-1 adrenergic agonist mitigates unloading-induced bone loss by maintaining formation. Med Sci Sports Exerc 45: 1665–1673. 23470310 10.1249/MSS.0b013e31828d39bc

[21] Swift JM, Nilsson MI, Hogan HA, Sumner LR, Bloomfield SA (2010) Simulated resistance training during hindlimb unloading abolishes disuse bone loss and maintains muscle strength. J Bone Miner Res 25: 564–574. 19653816 10.1359/jbmr.090811

[22] Parfitt AM, Drezner MK, Glorieux FH, Kanis JA, Malluche H, et al (1987) Bone histomorphometry: standardization of nomenclature, symbols, and units. Report of the ASBMR Histomorphometry Nomenclature Committee. J Bone Miner Res 2: 595–610. 3455637 10.1002/jbmr.5650020617

[23] Swift JM, Swift SN, Nilsson MI, Hogan HA, Bouse SD, et al (2011) Cancellous bone formation response to simulated resistance training during disuse is blunted by concurrent alendronate treatment. J Bone Miner Res 26: 2140–2150. 21509821 10.1002/jbmr.407

[24] Kondo H, Nifuji A, Takeda S, Ezura Y, Rittling SR, et al (2005) Unloading induces osteoblastic cell suppression and osteoclastic cell activation to lead to bone loss via sympathetic nervous system. J Biol Chem 280: 30192–30200. 15961387 10.1074/jbc.M504179200

[25] Takeda S, Elefteriou F, Levasseur R, Liu X, Zhao L, et al (2002) Leptin regulates bone formation via the sympathetic nervous system. Cell 111: 305–317. 12419242 10.1016/s0092-8674(02)01049-8

[26] Bouxsein ML, Devlin MJ, Glatt V, Dhillon H, Pierroz DD, et al (2009) Mice lacking beta-adrenergic receptors have increased bone mass but are not protected from deleterious skeletal effects of ovariectomy. Endocrinology 150: 144–152. 18801900 10.1210/en.2008-0843 PMC2630907

[27] Dufour C, Holy X, Marie PJ (2007) Skeletal unloading induces osteoblast apoptosis and targets alpha5beta1-PI3K-Bcl-2 signaling in rat bone. Exp Cell Res 313: 394–403. 17123509 10.1016/j.yexcr.2006.10.021

[28] Elefteriou F, Ahn JD, Takeda S, Starbuck M, Yang X, et al (2005) Leptin regulation of bone resorption by the sympathetic nervous system and CART. Nature 434: 514–520. 15724149 10.1038/nature03398

[29] Pierroz DD, Bonnet N, Bianchi EN, Bouxsein ML, Baldock PA, et al (2012) Deletion of beta-adrenergic receptor 1, 2, or both leads to different bone phenotypes and response to mechanical stimulation. J Bone Miner Res 27: 1252–1262. 22407956 10.1002/jbmr.1594

[30] Sakata T, Halloran BP, Elalieh HZ, Munson SJ, Rudner L, et al (2003) Skeletal unloading induces resistance to insulin-like growth factor I on bone formation. Bone 32: 669–680. 12810174 10.1016/s8756-3282(03)00088-7

[31] Sakata T, Wang Y, Halloran BP, Elalieh HZ, Cao J, et al (2004) Skeletal unloading induces resistance to insulin-like growth factor-I (IGF-I) by inhibiting activation of the IGF-I signaling pathways. J Bone Miner Res 19: 436–446. 15040832 10.1359/JBMR.0301241 PMC10720400

[32] Kellenberger S, Muller K, Richener H, Bilbe G (1998) Formoterol and isoproterenol induce c-fos gene expression in osteoblast-like cells by activating beta2-adrenergic receptors. Bone 22: 471–478. 9600780 10.1016/s8756-3282(98)00026-x

[33] Kondo H, Takeuchi S, Togari A (2013) beta-Adrenergic signaling stimulates osteoclastogenesis via reactive oxygen species. Am J Physiol Endocrinol Metab 304: E507–515. 23169789 10.1152/ajpendo.00191.2012

[34] Moore RE, Smith CK 2nd, Bailey CS, Voelkel EF, Tashjian AH Jr (1993) Characterization of beta-adrenergic receptors on rat and human osteoblast-like cells and demonstration that beta-receptor agonists can stimulate bone resorption in organ culture. Bone Miner 23: 301–315. 7908582 10.1016/s0169-6009(08)80105-5

[35] Togari A, Arai M, Mizutani S, Koshihara Y, Nagatsu T (1997) Expression of mRNAs for neuropeptide receptors and beta-adrenergic receptors in human osteoblasts and human osteogenic sarcoma cells. Neurosci Lett 233: 125–128. 9350848 10.1016/s0304-3940(97)00649-6

